# Optimizing Small RNA Sequencing for Salivary Biomarker Identification: A Comparative Study of Library Preparation Protocols

**DOI:** 10.3390/ijms262311437

**Published:** 2025-11-26

**Authors:** Ulrike Kegler, Nathalie Ropek, Manuela Hofner, Silvia Schönthaler, Klemens Vierlinger, Christa Nöhammer

**Affiliations:** Competence Unit Molecular Diagnostics, Center for Health & Bioresources, Austrian Institute of Technology GmbH, Giefinggasse 4, 1210 Vienna, Austria; ulrike.kegler@ait.ac.at (U.K.);

**Keywords:** microRNA, saliva, plasma, extracellular vesicles, next generation sequencing, library preparation

## Abstract

MicroRNAs (miRNAs) are small non-coding RNAs that regulate gene expression and hold significant potential as biomarkers. Saliva, a non-invasive and easily accessible biofluid, offers a promising alternative to blood for miRNA-based diagnostics. However, miRNA profiling by next-generation sequencing (NGS) is highly influenced by library preparation protocol, which can introduce detection and quantification biases. This study compared four commercial small RNA library preparation kits—QIASeq miRNA library kit (Qiagen), RealSeq-Biofluids Plasma/Serum miRNA library kit (Somagenics), Small RNA-seq library prep kit (Lexogen) and NEBNext multiplex small RNA library prep set for illumina (set 1) (New England BioLabs)—to evaluate their performance in profiling miRNAs from cell-free saliva, plasma and their extracellular vesicles (EVs). Using both synthetic reference and biological samples, we assessed the kits’ efficiency in handling low RNA input, minimizing bias and detecting diverse miRNAs. QIAseq outperformed the others, showing the highest miRNA mapping rates, minimal adapter dimers and the broadest miRNA detection, particularly in saliva. Moreover, substantial overlap between saliva- and plasma-derived miRNAs supports saliva’s diagnostic potential. Overall, this study underscores the critical impact of library preparation on miRNA sequencing outcomes and offers guidance for selecting optimal protocols for biomarker discovery from non-invasive sample matrices.

## 1. Introduction

MicroRNAs (miRNAs) are small non-coding RNAs (~22 nucleotides) with a regulatory function to post-transcriptionally influence gene expression by interaction with messenger RNAs (mRNAs), thereby inhibiting their translation [1]. miRNAs have been shown to impact biological processes in healthy and diseased individuals and can be found in tissue as well as biofluids including plasma, serum, urine or even saliva [2,3]. Saliva is a widely unexplored body fluid but is most attractive for diagnostic/medical application and use. Unbeatable advantages like non-invasiveness, easy accessibility and its almost unlimited source strengthen the suitability for diagnostics using biomarkers [4,5,6]. Besides DNA and proteins, miRNA can be present inside small extracellular vesicles (sEVs). These freely circulating vesicles are surrounded by a membrane-protein-containing lipid bilayer and have a typical size smaller than 200 nm [7,8]. EVs hold a tremendous potential for diagnostics because they play an important role in cell-to-cell communication in both health and disease. In addition, extracellular vesicles are constantly secreted by cells and mirror the condition of their cell of origin [9].

Next generation sequencing (NGS) has evolved to be a powerful tool for detecting and measuring miRNA expression; moreover, it also allows the discovery of novel miRNA species in contrast to quantitative PCR (qPCR) and microarray platforms [10]. NGS further allows single base resolution and became cost-efficient as it enables multiplexing of samples. Numerous manufacturers developed specific approaches of NGS library preparation suitable for small RNA and, in particular, miRNA profiling. A typical library preparation protocol consists of (i) the binding of adapter sequences to the miRNAs, (ii) reverse transcription, (iii) PCR amplification and (iv) library amplicon clean-up. All these steps can cause biases with respect that certain miRNAs that are preferably detected compared to other miRNA species [11,12,13,14]. This leads to a skewed representation where some miRNAs are overrepresented, and others are underrepresented or even remain undetected.

In this study, we comprehensively compared four commercially available small RNA library preparation kits: (a) QIASeq miRNA library kit (Qiagen), (b) RealSeq-Biofluids Plasma/Serum miRNA library kit (Somagenics), (c) Small RNA-seq library prep kit (Lexogen) and (d) NEBNext multiplex small RNA library prep set for illumina (set 1) (New England BioLabs). We tested the performance of these kits in terms of their biological and technical variability. For demonstrating biological feasibility, we prepared libraries from biofluids and compared the following sample matrices: (i) plasma (minimal invasive) vs. saliva (non-invasive) and (ii) cell-free saliva and plasma vs. saliva- and plasma-derived extracellular vesicles. We particularly tested here the applicability of saliva for biomarker discovery to lay the foundation for transferring diagnostics from a minimally invasive approach using biomarkers from blood to a non-invasive approach applying salivary biomarkers instead. The technical reproducibility was checked by (i) testing each biological sample in duplicate and (ii) using a synthetic miRNA miRXplore Universal Reference (Miltenyi Biotec). We investigated the different library preparation kits for their performance, especially in respect of handling low RNA input concentrations, different sources of biofluids, as well as miRNA representation. To our best knowledge, this is the first study comparing a set of different library preparation approaches in cell-free saliva and saliva-derived EVs.

## 2. Results

### 2.1. Experimental Overview of Small RNA Library Preparation Techniques

**Figure 1:**
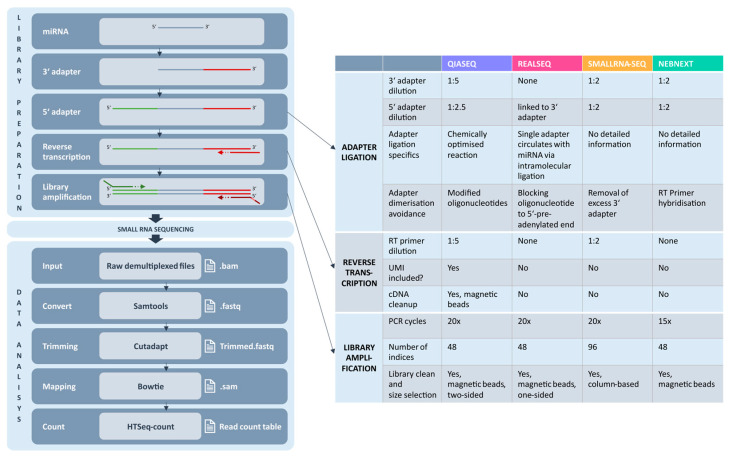
Overview and comparison of small RNA sequencing library preparation approaches. On the left, a general summary of the library preparation steps, which are similar for all kits, is given. In the table on the right, QIAseq miRNA library kit (purple, QIASeq) (Qiagen), RealSeq-Biofluids Plasma/Serum miRNA library kit (magenta, Realseq) (Somagenics), Small RNA-seq library prep kit (orange, smallRNA-Seq) (Lexogen), and NEBNext multiplex small RNA library prep set for Illumina (green, NEBNext) (New England BioLabs) are compared regarding the three major differences in (i) adapter ligation, (ii) reverse transcription, and (iii) library amplification including multiple sub-aspects for each category. After small RNA sequencing, the raw data was used as an input to a computational pipeline which is illustrated on the bottom left.

For the present comparison study of small RNA library kits, we investigated not only cell-free (cf) saliva and plasma, but also derived extracellular vesicles (EVs). For quality control, we further used miRXplore Universal Reference (130-093-521, Miltenyi Biotec, Bergisch Gladbach, Germany) in parallel for all investigated library preparation kits. Mature miRNAs, possessing 3′ hydroxyl group and a 5′ phosphate group, were first extracted and concentrated from the sample matrices studied by a defined method (miRNeasy Serum/Plasma advanced kit, 217204, Qiagen, Hilden, Germany), which allows isolation of all types of RNA including miRNAs. Small RNA library preparations started off with sequentially ligating the 3′ adapters and 5′ adapters to the corresponding miRNA ends. The adapters built the basis for subsequently performing a universal reverse transcription. The generated cDNA was then multiplied by PCR amplification, which introduced the barcodes for multiplexing samples and the sequencing index primers. After completing library amplification, obtained amplicons were cleaned up for removal of PCR components often combined with size selection for removal of long library fragments and adapter-dimers. The key steps of the library preparation workflow are illustrated in Figure 1.

The manufacturers of the library preparation kits developed different strategies to overcome quantification bias, adapter dimerization and inefficient size selection. As implemented in the table of Figure 1, for QIASeq miRNA library kit (QIASeq) (Qiagen), RealSeq-Biofluids Plasma/Serum miRNA library kit (Realseq) (Somagenics), Small RNA-Seq library prep kit (small RNA-Seq) (Lexogen), and NEBNext multiplex small RNA library prep set for illumina (NEBNext) (New England BioLabs) different adapter dilutions are recommended. The main differences between the library preparation kits concern the adapter structure and their ligation to the miRNA. In the case of QIASeq, small RNA-Seq and NEBNext two adapters, a 3′ and a 5′ adapter, are ligated to the respective ends of each miRNA. Especially for QIASeq, this comprises a chemically optimized reaction. RealSeq has another approach, applying only one single adapter where 3′ adapter and 5′ adapter are combined and then using a circularized miRNA-adapter construct as template for RT reaction. For preventing adapter dimerization, QIASeq uses modified oligonucleotides, whereas RealSeq blocks adapter dimerization before miRNA-adapter circularization. The small RNA-Seq from Lexogen removes excess 3′ adapter before attaching the 5′ adapter. In contrast, NEBNext applies RT primer hybridization to avoid negative effects caused by adapter dimerization. As far as the reverse transcription and library amplification steps are concerned, there are only small variations in RT primer dilutions and PCR cycle numbers for the amplification between the studied kits. All kits include a final size selection step. QIASeq, RealSeq and NEBNext utilize magnetic beads for this purpose. Realseq and NEBNext perform a single-round, one-sided purification to remove fragments larger than the target amplicon. In contrast, QIASeq applies a more sophisticated two-sided selection, using magnetic beads to eliminate both smaller and larger unwanted fragments for improved amplicon cleanup. Unlike the other kits, the Small RNA-Seq kit uses a column-based approach for size selection.

Processed libraries are finally subjected to quality control (QC) and adjusted to a distinct library concentration. To pass QC, peaks in the electropherogram of the fragment analyzer run should appear around 150–160 bp as this represents the miRNA library. Peaks arising at 120–130 bp represent the unwanted adapter-dimers. For QIASeq, due to a different adapter structure, the miRNA library peaks show up at around 180 bp, whereas the 160 bp peak represents the adapter-dimers (Supplementary Figure S1). After receiving raw sequencing data, data analysis was performed applying specific bioinformatic tools as illustrated in Figure 1.

### 2.2. Quantitative Assessment of miRNA Representation During Small RNA Library Preparation

The miRXplore universal reference (Miltenyi Biotec) contains 998 equimolar synthetic miRNAs [15]. This character of the reference makes it suitable for checking whether each of the miRNAs is equally amplified along library preparation and identical sequencing read counts are finally obtained for every miRNA. The miRNA reference has been validated via various spike-in experiments by Miltenyi Biotec [16] and was applied in several microarray and RNA sequencing studies. The miRXplore reference contains 564 human miRNAs within a mixture of a total of 998 equimolar miRNAs, including mouse, rat and viral miRNA sequences. The small RNA-Seq kit (Lexogen) had to be excluded from the miRNA reference analysis due to extremely low read counts. The three remaining library preparation kits were included in the bioinformatic analysis workflow to determine the performance of each library preparation with respect to reliable and balanced miRNA detection. For each kit, an almost identical total number of miRNAs was detected.

**Figure 2:**
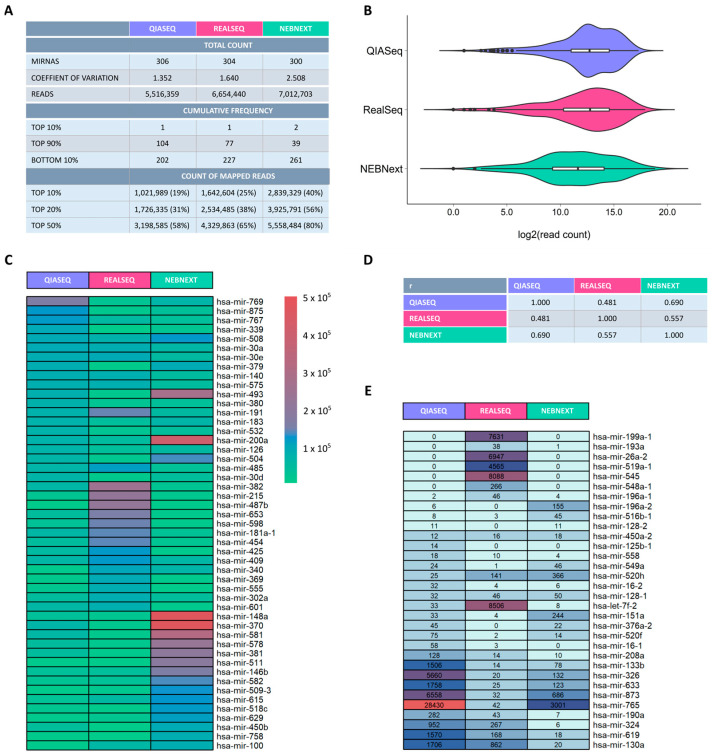
Differences in small RNA sequencing libraries kits when applying the miRXplore universal reference. Four small RNA sequencing library preparation kits were tested in parallel with the miRXplore universal reference (Miltenyi Biotec) containing 998 equimolar miRNAs out of which 564 are human. As the Small RNA-seq library prep kit (Lexogen) gave only very low overall read counts, in the end, only three kits were included in the comparative analysis (**A**) The table summarizes the overall detected miRNAs per kit, the kit-specific coefficient of variation (standard deviation (SD) divided by the absolute value of the mean), the cumulative frequency, as well as the overall mapped read counts of the top10, top20 and top50 miRNAs. Only miRNAs with a read count > 10 were considered. (**B**) Violin plots to demonstrate the frequency and distribution of miRNA counts. (**C**) Heatmap with the 20 most abundant miRNAs per kit shows the differences in miRNA-specific reads counts and highlights the preferentially detected miRNA species for each library kit. (**D**) Correlation analysis of read counts using the Spearman correlation coefficient (r). (**E**) 20 miRNAs with the lowest read counts within each library preparation kit are listed and composed in a heatmap to illustrate the differences in read counts in the bottom range and to highlight uniquely detected miRNAs per library preparation kit.

Most synthetic miRNAs were detected by QIASeq with a total count of 306, followed by RealSeq (304 miRNAs) and NEBNext (300 miRNAs). Due to the fact of equal frequency of each miRNA in the reference, all detected miRNAs are assumed to be sequenced with equal read counts. To control the variability, the coefficient of variation (CV), defined by the ratio of standard deviation to the mean of read counts, was calculated. With a CV of ~1.4, QIASeq showed the lowest variation. RealSeq was comparable to QIASeq (CV~1.6). In contrast, NEBNext seemed to be more variable with a CV of ~2.5. Also, the cumulative frequency for Top10%, Top90% and Bottom10% as well as the count of mapped reads for Top10%, Top20% and Top50% indicated that more miRNA species with similar counts could be detected using QIASeq than with RealSeq and NEBNext (Figure 2A). The distribution of log2-transformed read counts is shown as violin plots to indicate any imbalanced abundance of miRNAs (Figure 2B). The integrated boxplots show the mean and the interquartile range. It can be seen that the read counts are more condensed around the mean and the interquartile range in QIASeq, which indicates the most equimolar distribution of read counts in comparison to the other library preparation kits. RealSeq and NEBNext show a wider range of read counts and a more dispersed distribution along the range, which means that there are both very high and very low read counts for different miRNAs which contradicts the given equimolarity of miRNAs in the universal reference sample. Furthermore, we calculated the correlation of mean read count for each library preparation kit with the other protocols using the Spearman correlation coefficient (r), which indicated weak correlation (ranging from 0.481 to 0.690) (Figure 2D). To explore more details on the capabilities of the kits with respect to reliable miRNA detection, the miRNA species with the lowest and highest read counts obtained by each methodology was examined. Some miRNAs did not come up using QIASeq but appeared in one of the other kits and vice versa (Figure 2E). Even more interestingly, the most abundant miRNA species from one kit could also be found with the other techniques but there were huge differences in the read count numbers (Figure 2C).

### 2.3. Distribution of Non-Coding RNA Categories in Cell-Free Saliva and Plasma and the EVs Derived from Them

**Figure 3:**
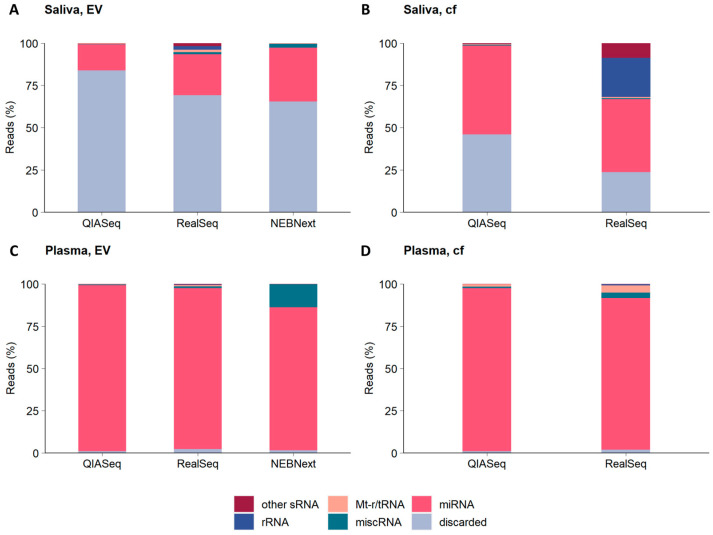
Categories of non-coding small RNAs detected in cell-free saliva and plasma and thereof derived EVs. Bar plots showing the mean percentages of mapped small RNA reads for various non-coding RNA subtypes for (**A**) saliva extracellular vesicles (EVs); (**B**) saliva cell-free (cf); (**C**) plasma EVs and (**D**) plasma cf. The mapped reads were referred to the following non-coding (nc) RNA classes: rRNA, mt-r/tRNA, miscRNA, miRNA and other sRNA (including piRNA, rRNA, siRNA, snRNA, snoRNA, tRNA and vaultRNA). Mapped reads that did not align to ncRNA were classified as “discarded”.

For the second part of our study, we applied all four library preparation kit protocols not only on cell-free saliva and plasma samples from healthy volunteers, but also on thereof isolated extracellular vesicles (EVs). The small RNA-Seq kit (Lexogen) had to be excluded from this analysis and comparison study as only very low read counts could be obtained after sequencing. Sequencing-detected mapped reads were sorted into six categories and types of non-coding (nc) RNAs as follows: (i) microRNA (miRNA); (ii) miscellaneous RNA (miscRNA); (iii) mitochondrial transfer and ribosomal RNA (Mt-t/rRNA); (iv) ribosomal RNA (rRNA); (v) other small RNA (sRNA) (including small nucleolar RNA (snoRNA), small nuclear RNA (snRNA) and small cajal body-specific RNA (scaRNA)) and (vi) discarded reads. Reads were classified as discarded because they mapped to genomic regions other than small ncRNA genes in the human genome (Figure 3). Beside these biotype groups, there are no mapped reads sorted into the classes of piwi-interacting RNA (piRNA), small-interfering RNA (siRNA) and vaultRNA, which are also ensembl biotype small ncRNA annotation subgroups [17,18].

The proportions of mapped reads aligned to miRNAs genes for each saliva sample type as follows: QIASeq (15.32% in saliva EVs; 52.42% in cf saliva), RealSeq (24.26% in saliva EVs; 43.18% in cf saliva) and NEBNext (31.84% in saliva EVs; not measured in cf saliva) (Figure 3A,B and Figure S2). For each plasma sample type, the proportions of mapped reads aligned to miRNA genes were as follows: QIASeq (98.33% in plasma, EV; 96.54% in plasma, cf), RealSeq (95.22% in plasma, EV; 89.82% in plasma, cf) and NEBNext (84.64% in plasma, EV; not measured in plasma, cf) (Figure 3C,D and Figure S2). Comparing the kit data for each sample type separately, the mapped miRNA rate was quite similar. Examining the cell-free plasma and its extracellular vesicles, QIASeq presented a higher miRNA enrichment compared to RealSeq and NEBNext. Interestingly, RealSeq obtained the same percentage of miRNA in plasma EVs but not in cf plasma in comparison to QIASeq. For saliva extracellular vesicles, a declined miRNA percentage was observed compared to RealSeq and NEBNext. QIASeq detected the highest miRNA proportion from cell-free saliva. Of note, miRNA portions were higher in plasma (92.91% on average) in contrast to saliva (33.40% on average) obtained with all techniques.

In saliva samples, a substantial amount of reads from the libraries were discarded because they did not map to any small RNA gene of the human genome (Figure 3). Since only mapped reads were sorted for the small RNA composition, these reads are probably related to other gene biotype groups such as protein-coding genes. Especially for salivary EVs, the discarded read percentage of ~65–83% was extremely high. On the other hand, the eliminated reads in plasma libraries could be disregarded. In more detail, in QIASeq, only 1% of the mapped reads were discarded, which is lower than in RealSeq and NEBNext (Supplementary Figure S2). In summary, Qiaseq led to highest proportion of miRNAs (both in total read count and percentage) and showed the lowest percentage of discarded reads.

In addition to miRNA, other small RNA subclasses were also discovered. Most other small RNA species were detected by RealSeq. The composition shift in RealSeq libraries could be based on the different adapter ligation technologies. Interestingly, the rRNA was only captured by RealSeq.

### 2.4. miRNA Profile Characteristics in Saliva and Plasma

**Figure 4:**
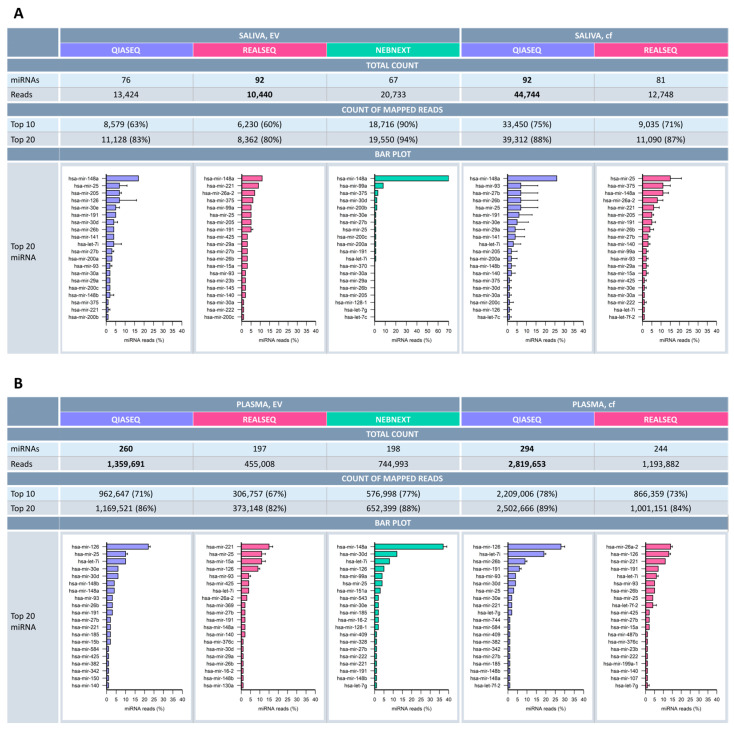
miRNA profile characteristics in saliva and plasma samples. Summary of the 20 most abundant miRNAs detected via the various small RNA library kits (QIASEQ, REALSEQ, NEBNEXT) in human saliva EVs and cell-free saliva (**A**) and human plasma and plasma-derived EVs (**B**). The table shows the total number of miRNAs and their corresponding read counts, as well as the read count for the top10 and top20 most abundant miRNAs in terms of percentage with respect to the total reads. Bar charts indicate the percentage of miRNA reads (%) associated with the top20 miRNA plotted per kit and biological sample type.

Potential differences in detected miRNA profiles were investigated in-depth not only by comparing the three different library preparation kits (QIASeq, RealSeq and NEBNext) but also studying the various sample matrices, namely cell-free saliva and plasma, as well as thereof derived extracellular vesicles. Here not only was the spectrum of detected miRNA species per kit and sample matrix explored, but also the overall number of detectable miRNAs. Additionally, the mapped miRNAs were ranked according to their ascending number of reads. Regarding the total miRNA species, about 15–30% more distinct miRNAs could be detected in all sample matrices using the QIASeq kit with the exception of salivary EVs (Figure 4). Moreover, obtained overall miRNA read counts were highest with QIASeq (Figure 4). In the table of Figure 4, cumulative frequency of reads accounting for the Top10 and Top20 most abundant miRNAs is listed. Specific miRNA species were differentially well-detected, depending on which method was applied. Overall difference in detected miRNA types and expression levels were observed to be dependent on which library approach was used. This is indicated by the high percentage of reads attributed to only the Top10 and even more to the Top20 most abundant miRNAs. In other words, only a few miRNAs account for the majority of the total read count. For QIASeq, 63% of the reads were already accounted to the Top10 miRNAs in salivary EVs and 71% in plasma EVs. For cell-free samples of both matrices, the percentage was even slightly higher. As shown in the bar charts, where the mean percentage of reads associated with the 20 most abundant miRNAs is illustrated in descending order, the miRNA profile differed such that each kit brought up different miRNAs. Looking deeper at the distinct miRNA species, it became evident that about 50% within the Top20 miRNAs were shared between the library prep methodologies (Figure 4 and Figure S3).

**Figure 5:**
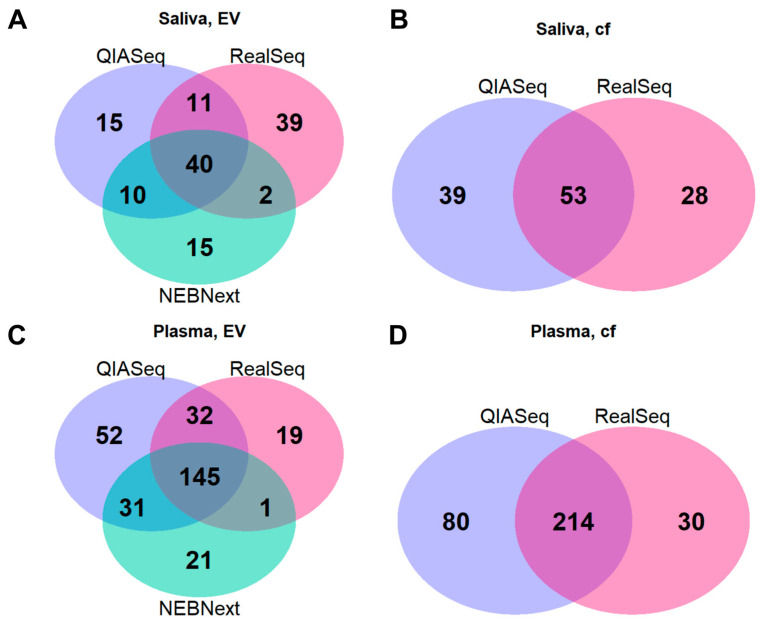
Overlaps of miRNA profiles in salivary and plasma extracellular vesicles, as well as cell-free plasma and saliva. The overlap of all detected miRNAs between each library prep technique are illustrated using the Venn diagrams plotted for (**A**) saliva, extracellular vesicle (EV); (**B**) saliva, cell-free (cf); (**C**) plasma, EV and (**D**) plasma, cell-free (cf).

On the other hand, there were also some miRNA species which emerged specifically using a distinct methodology. Along these lines, the entire spectra of miRNAs detected by each library preparation kit were merged to elucidate the overlaps. Interestingly, significant overlaps could be found for all sample matrices investigated. For saliva, around 50 miRNAs could be found in both EV and cf samples comparing QIASeq and RealSeq. Including the NEBNext kit for saliva EVs, 40 miRNAs could still be detected with all preparation kits (Figure 5A,B). For plasma, both EVs and plasma per se, the overlaps were even greater with about 200 shared miRNAs when comparing QIASeq and RealSeq (Figure 5C,D). Of particular interest was the fact that QIASeq could detect by far the most unique miRNA species (Figure 5) in plasma and plasma-derived EVs.

**Figure 6:**
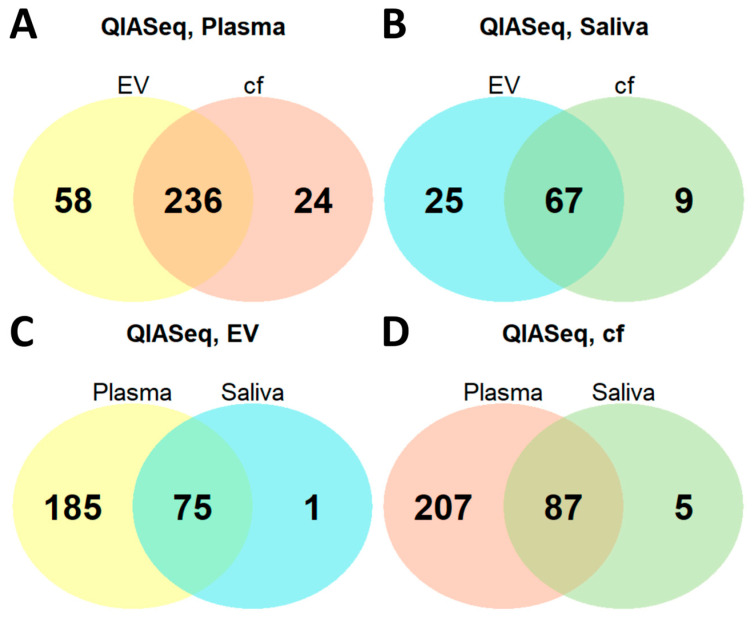
Overlaps of miRNA profiles with respect to sample matrices. The overlap of miRNAs between the different sample matrices detected by QIASeq is illustrated using Venn diagrams plotted for (**A**) QIASeq plasma, (**B**) QIASeq saliva, (**C**) QIASeq extracellular vesicle and (**D**) QIASeq cell-free.

We were also interested in the extent to which diagnostics from the minimally invasive sample matrix blood can be potentially translated to the totally non-invasive body fluid saliva. Taking the QIASeq kit as an example, we compared plasma and saliva, and explored whether an intersection of their miRNA profiles is given and how much miRNAs are shared between the two body fluids. The similarity of plasma and saliva is very high, which is emphasized by the fact that 75 out of 76 miRNA from salivary EVs were also detected in plasma EVs and 87 out of 92 miRNAs from cf saliva were found in plasma (Figure 6C,D). Given the fact that the EVs are extracted out of a cell-free sample, followed by extracellular vesicular nucleic acid isolation, we assumed miRNAs found in EVs should also be detected in cf samples. For plasma, 236 miRNAs were observed in both fractions; for saliva, 67 shared miRNAs were found. In contrary to our expectations, the miRNA numbers detected in EVs are higher than in the corresponding cell-free fluids (Figure 6A,B). This effect may be observed considering miRNAs could be concentrated since they are gathered in EVs. These miRNA species could be under the detection limit in the cell-free sample. The results of the described comparisons were comparable when RealSeq kit was applied instead of QIASeq (Supplementary Figure S4).

### 2.5. Sequencing Efficiency

**Figure 7:**
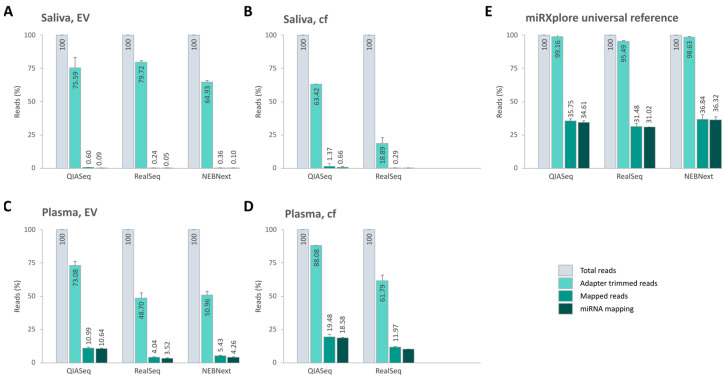
Summary of sequencing efficiency. The bar plots show the mean percentage of the reads after adapter trimming, genome and miRNA mapping, respectively, per library preparation kit. The sequencing efficiency for miRNAs is reflected by the overall miRNA reads after passing adapter trimming and mapping compared to the total read counts.

While running our computational analysis pipeline, miRNA reads were assessed and had to pass certain filters. Firstly, the adapters were trimmed, which means that reads with no adapter and too short reads (<15 bp) were removed. Following that, the reads were mapped to the GRCh38 human reference genome (Ensembl). Both all unmapped and multimapped reads were discarded (only single mapped reads are accepted). The passed reads are typically used for downstream data analyses. The proportion of passed reads relative to the overall total reads reflects the sequencing efficiency. Along these lines, the percentages of reads passing the various filters for all sample matrices, as well as the miRXplore reference, were calculated. As far the miRXplore reference is concerned, the sequencing efficiency for mapped reads as well as miRNAs was around 35% for all library techniques (Figure 7E). In plasma, the sequencing efficiency for miRNAs was only about 10% on average, which is given by the fact that miRNA is only a part of the RNA found in body fluids (Figure 7C,D). The sequencing efficiency for miRNAs in saliva samples decreased even more and became less than 1% (Figure 7A,B). Possible reasons for this can be bacterial RNA contamination, in addition to an overall low concentration of human small RNAs. For epigenetic biomarker discovery, the rate of miRNA mapped reads is highly relevant.

After combining the data for saliva, plasma, and the total, the proportion of the total reads mapping to human miRNA species was as follows: 0.38% (saliva)/14.61% (plasma)/7.5% (total) for QIASeq; 0.08% (saliva)/6.81% (plasma)/3.45% (total) for RealSeq; 0.10% (saliva)/4.26% (plasma)/2.18% (total) for NEBNext. In conclusion, using QIAseq, the highest percentage of miRNA reads could be detected (Supplementary Figure S5).

## 3. Discussion

In our study, we compared four commercially available library preparation kits specifically designed for small RNA analysis, with a focus on their ability to detect miRNAs. A key criterion in our evaluation was the kits’ performance with low sample input amounts, as well as challenging sample types, such as cell-free saliva and the thereof derived extracellular vesicles. The motivation for this study aligns with the overall goal of our research group to promote non-invasive diagnostics via saliva biomarkers as an alternative to minimally invasive blood-based diagnostics.

In recent years, next generation sequencing (NGS) has become the preferred method for miRNA biomarker discovery. However, NGS library preparation introduces various biases, such as adapter ligation inefficiency, adapter dimerization and inadequate library purification, which can distort miRNA abundance measurements, complicate the detection of miRNA as potential biomarkers and reduce the reproducibility of sequencing results [19]. Previous comparative studies have largely focused on sample matrices like plasma, serum, tissue or universal reference [20,21,22,23,24,25,26]. To our best knowledge, this is the first study to simultaneously investigate cell-free saliva, plasma samples, their respective EVs, and a synthetic miRNA reference to compare four different small RNA library preparation techniques. In this way, the kits were specifically tested for their feasibility using saliva samples, because all the tested commercial kits are optimized for investigation of small RNAs from blood, cells or tissue.

### 3.1. Library Preparation and Adapter Strategies

The most critical challenge in small RNA library preparation is the adapter ligation step and the associated risk of unfavorable adapter dimerization, which is addressed and we tried to overcome this via different strategies by the evaluated kit manufacturers. QIASeq, small RNA-Seq and NEBNext use two sequentially ligated adapters to the 3′ and 5′ ends of miRNAs. For dimerization prevention, QIASeq implements modified oligonucleotides, whereas small RNA-Seq removes excess 3′ adapter before ligating 5′ adapter [27,28]. On one hand, NEBNext blocks dimer formation by RT primer hybridization to excess 3′ adapter [29]. In contrast, RealSeq incorporated both sequencing adapters into a single adapter, which is bound to miRNA and the product is then circularized via intramolecular ligation [24] (see Figure 1). All small RNA libraries offer gel-free cleanup for increased reproducibility. Their protocols offer a one-day protocol including similar hands-on times, which is compatible with high-throughput. If a large number of samples is processed, a fast and automated protocol is desirable. This is enabled by QIASeq and NEBNext as they support integration with automated liquid-handling robots.

While QIASeq provides magnetic beads for purification within the kit, NEBNext and RealSeq kits require them to be sourced separately. After cleanup and size selection, the library quality was assessed using the Agilent Fragment Analyzer system. Here different peak profiles from saliva and plasma were observed (Supplementary Figure S1). Clear, sharp peaks were observed for plasma-derived libraries, which allowed us to clearly distinguish the adapter-dimer peaks from the library specific peaks, whereas saliva-derived libraries showed slightly broader peak profiles. Overall, all kits generated size-appropriate library peaks and accordingly, sequencing could be successfully performed from all libraries. Importantly, the extent of formed adapter dimers has an impact as it results in contamination of the miRNA sequencing libraries, which strongly influences the sequencing depth and results in a reduction in the number of proper reads [19]. In our comparison, RealSeq and NEBNext both faced the issue of provoking adapter dimer formation. RealSeq displayed an approximately 50:50 ratio of adapter dimer to miRNA library peaks. QIASeq clearly outperformed the other library prep approaches by showing no visible adapter dimers, highlighting a clear advantage in library purity.

### 3.2. Sequencing Performance and Kit Exclusion

For analysis of sequencing data, the Small RNA-Seq kit yielded low read counts and high variability, leading to its exclusion from sequencing analysis. This may be due to the kit’s known incompatibility with multiplexing alongside other vendors’ libraries. However, a separate sequencing lane would have been required, which was not feasible for our study design and budget.

### 3.3. Comparative Analysis of Saliva vs. Plasma

Among the successfully sequenced libraries, distinct differences in small RNA composition were observed between saliva and plasma (see Figure 3). Other significant differences between the two body fluids comprised overall miRNA reads and number of discarded reads. Plasma libraries exhibited approximately three times more miRNA species than saliva (e.g., for QIASeq 260–294 vs. 76–92; see Figure 4). The portion of discarded reads especially in the saliva EV samples was about 65–83% (see Supplementary Figure S2).

### 3.4. miRNA Detection and Biomarker Relevance

All kits detected a wide range of miRNAs from the miRXplore universal reference, which contains 558 human-associated miRNAs out of 998 miRNAs in equimolar concentrations (see Figure 2). Despite equal concentrations of synthetic miRNAs present in the reference, bias in relation to true miRNA species abundances was evident for all libraries. For biomarker discovery, the priority is the breadth of miRNA species detected rather than the total read count. Each kit identified different sets of miRNAs, with distinct ranking patterns based on read abundance. The most essential finding was that QIASeq detected the highest number of unique miRNA species across most sample types, reinforcing its utility in broad-spectrum biomarker screening. Nevertheless, QIASeq performance was slightly lower in saliva-derived EVs (see Figure 5), compared to all other sample types.

Interestingly, at least 94% of saliva miRNAs were also detected in plasma, supporting the feasibility of saliva as a substitute for blood in miRNA-based diagnostics. However, plasma still yielded ~2.5 times more unique miRNA species (see Figure 6). Nonetheless, EVs showed greater miRNA diversity than cf samples, likely due to concentration of miRNAs within vesicles.

### 3.5. Library Quality and Mapping Efficiency

A key determinant of kit performance is the percentage of sequencing reads that map to known miRNAs. QIAseq libraries had the highest miRNA mapping rates for both plasma and saliva (see Figure 4). While all kits performed comparably with the synthetic reference sample, saliva consistently yielded low miRNA mapping percentages across all kits, likely due to its microbial complexity and low endogenous miRNA concentration. Future studies should explore whether non-aligned reads correspond to microbial or novel RNA species.

### 3.6. UMIs and Quantification Accuracy

Besides the crucial step of adapter ligation, the clonal PCR amplification-related enrichment can also introduce bias in small RNA sequencing. Here, QIASeq incorporates unique molecular indices (UMIs), which are a short random sequence introduced during reverse transcription reaction to distinguish true biological duplicates from PCR artifacts [30]. This enables more accurate quantification of miRNA expression. However, UMIs were not used in RealSeq or NEBNext, limiting our ability to compare absolute miRNA quantities across all kits (see Figure 1). Further investigation of UMI utility is recommended.

## 4. Materials and Methods

### 4.1. Sample Collection and Preprocessing

Saliva and plasma samples were donated by healthy volunteers (all working at the Austrian Institute of Technology) and judged by the local ethics committee of the city of Vienna to decide if ethical approval is required if the intended use comprises molecular method evaluation and optimization. Whole saliva was collected from seven healthy individuals (three males, four females) by unstimulated spitting into a sterile 50 mL Falcon tube. Saliva donors were not allowed to smoke, eat or drink one hour before saliva donation. Ten minutes before starting collection, each donor had to rinse his/her mouth with water without swallowing it. During the spitting process, eating and drinking were not allowed. After a minimum of 15 mL saliva per donor was collected, samples were centrifuged at 3000× *g* and 4 °C for 20 min and cell-free supernatant of each sample was carefully transferred and pooled into a sterile glass bottle without interrupting the cell pellet. Aliquots of 1 mL cell-free saliva were stored at −80 °C. Blood was drawn from 10 healthy participants (five males, five females) using the Vacuette tube K3E K3EDTA, 9 mL (455036, Greiner Bio-One, Kremsmünster, Austria). After mixing by gently inverting, plasma was immediately separated via centrifugation at 2000× *g* and room temperature for 20 min, but processed within 1 h. After pooling, aliquots of 1 mL cell-free plasma were stored at −80 °C until further processing.

### 4.2. Extracellular Vesicle Isolation

For extracellular vesicle isolation, miRCURY Exosome Isolation kit—Serum and Plasma (76603, Qiagen, former Exiqon, Hilden, Germany) was applied according to the manufacturer’s instructions with adjustments made to the starting volume and minor modifications corresponding to this change. The starting input volume was 1.5 mL cell-free saliva or plasma. Precipitated EVs, also derived from 1.5 mL original body fluid, were resuspended in 300 µL 1× TBS before RNA was isolated. Each method was tested in duplicate.

### 4.3. EDTA Removal from Plasma EVs

The enzyme inhibitor EDTA was removed from the plasma samples by using the Amicon Ultra-0.5 centrifugal filter devices (UFC503096, Milipore, Burlington, VT, USA) according to the manufacturer’s manual. Afterwards, EVs were washed twice with 300 µL 1× TBS (pH 7.4) and finally resuspended in 300 µL 1× TBS (pH 7.4).

### 4.4. DNase and RNase Treatment of EVs

The EVs that were isolated and resuspended in 300 µL 1× TBS were treated with 8 µL Dnase 1, RNase-free (1 U/µL) (EN0521, Thermo Fisher Scientific, Waltham, MA, USA) and 3 µL of RNase A, DNase and Protease-free (10 µg/µL) (EN0531, Thermo Fisher Scientific, Waltham, MA, USA). For the activity of the DNase 1, 30 µL of the 10× reaction buffer with MgCl_2_ (B43, Thermo Fisher Scientific, Waltham, MA, USA) was also added to the reaction. The reaction was incubated for 1 h at 37 °C with light shaking at 300 rpm.

### 4.5. RNA Isolation from EVs, Plasma and Cell-Free Saliva

The miRNeasy Serum/Plasma advanced kit (217204, Qiagen, Hilden, Germany) was applied to isolate RNA, including miRNA from the different sample types. For EV samples from saliva and plasma, the 341 µL DNase/RNase-treated EV suspension was combined with one third of the volume of the buffer PRL. Proteins were precipitated via mixing with one tenth of the original volume of the buffer RPP. The following steps of loading RNA and washing were handled according to the manufacturer’s instruction. For cell-free saliva and plasma samples, the starting volume was set to 500 µL. The samples were treated with the same buffer proportions as the EV samples and according to the manufacturer’s manual. All samples were eluted in 20 µL DNase/RNase-free water and stored at −80 °C.

### 4.6. Quantification of Total RNA Amount

The amount of total RNA was measured using the Quant-iT RiboGreen RNA kit (R11490, Thermo Fisher Scientific, Waltham, MA, USA) and the fluorospectrometer device NanoDrop 3300 (Thermo Fisher Scientific, Waltham, MA, USA), following the producer’s manual.

### 4.7. Small RNA Library Preparation for Sequencing

For the comparison study, four different library preparation kits for small RNA were originally tested: Small RNA-seq library prep kit (058, Lexogen, Vienna, Austria), NEBNext multiplex small RNA library prep set for Illumina (set 1) (E7300, New England BioLabs, Ipswich, MA, USA), QIASeq miRNA library kit (331505, Qiagen, Hilden, Germany), RealSeq-Biofluids Plasma/Serum miRNA library kit (600-00024, Somagenics, Santa Cruz, CA, USA). The starting input concentration was 5 ng RNA per reaction of each kit. All kits were performed following the vendors’ handbooks.

### 4.8. Quality Control of Library Amplicons

The quality control of the small RNA library was performed via HS NGS fragment kit (1–6000 bp) (DNF-474, Agilent, Santa Clara, CA, USA) on the 5300 Fragment Analyzer system (Agilent, Santa Clara, CA, USA) according to the manufacturer’s instructions. For the measurement the FA 48-capillary array short (33 cm, A2300-4850-3355, Agilent, Santa Clara, CA, USA) was installed. The samples were diluted 1:10 or 1:100, respectively, to fit the concentration range of the kit. The smear analysis was set to range from 100 to 300 bp.

### 4.9. Small RNA Sequencing

The concentration of the quantified library amplicons was adjusted to an equimolar range of 5 nM. Per sequencing lane, a mixture of a maximum of 20 samples was applied. The samples were sequenced via Illumina Hiseq 2500, applying 50 bp single reads (SR 50).

### 4.10. Statistical Analysis

The overall quality of the raw fastq files containing single end reads was determined with FastQC (version 0.11.8, [31]). A pipeline was set up to assure a reproducible analysis workflow and each dataset was analyzed in parallel (Figure 1). Adapter trimming at the 3′ end was performed with the respective adapters as recommended for each library kit by using Cutadapt (version 2.5, [32]) and allowing a 10% error rate. Additionally, short reads with <15 bp were removed. It must be mentioned that the unique molecular indices (UMIs), which are special for the QIASeq kit, are not included in a 50 bp single read protocol [27]. Trimmed and filtered reads were mapped with Bowtie (version 1.2.3, [33] to the indexed human reference GRCh38 from Ensembl) (ftp://ftp.ensembl.org/pub/release-98/fasta/homo_sapiens/dna/Homo_sapiens.GRCh38.dna.primary_assembly.fa.gz, accessed on 13 December 2019). The parameters used allowed one mismatch (−v 1), returned only one alignment with the best alignment score (--best --strata) and suppressed multimapping (−m 1). The obtained SAM files were transformed into BAM files and sorted with SAMtools (version 1.9, [34]). HTSeq-count (version 0.11.2, [35]) with the parameters -s no and --mode intersection non_empty used with the Ensembl reference GTF file (ftp://ftp.ensembl.org/pub/release-98/gtf/homo_sapiens/Homo_sapiens.GRCh38.98.chr.gtf.gz, accessed on 13 December 2019) to annotate the unique reads and obtain a read count table for each sample. The DESeq2 R package (version 1.24.0, [36]) was used to import the data and an exploratory analysis of the raw read counts was performed in R. miRNAs with a read count ≤ 10 per sample were excluded. A list of human miRNAs, represented in the miRXplore universal reference, was provided by Miltenyi Biotec.

Plots were generated in R 3.6.1 with the use of ggplot2 (version 3.2.1, [37]), pheatmap (version 1.0.12, [38]) and VennDiagram (version 1.6.20, [39]) packages.

## 5. Conclusions

All tested protocols successfully generated libraries from low-input RNA derived from diverse biofluids. However, miRNA yield and mapping efficiency varied significantly by kit and sample type. The choice of library preparation method should therefore be guided by the biological source, RNA quantity and research objectives.

Among the evaluated methods, QIAseq emerged as the most robust and versatile. It consistently produced high-quality libraries with minimal adapter dimers, superior miRNA enrichment and broad miRNA species detection, even in challenging matrices such as cell-free saliva and salivary EVs. Its optimized ligation chemistry, integrated clean-up steps and automation compatibility make it especially well-suited for high-throughput biomarker discovery efforts.

Ultimately, the success of small RNA sequencing depends heavily on the quality of the library preparation. While kit-specific biases may influence quantification, careful study design, particularly parallel processing of case and control samples, can reduce these effects. Deeper sequencing or kit selection tailored to sample complexity may further enhance miRNA detection. Given the growing interest in NGS for miRNA biomarker discovery, continued refinement of library preparation protocols will be essential.

## Data Availability

The data presented in this study are available on request from the corresponding author due to the intention to file a patent.

## Supplementary Materials

The following supporting information can be downloaded at https://www.mdpi.com/article/10.3390/ijms262311437/s1.

## Abbreviations

The following abbreviations are used in this manuscript:

cf	Cell-free
EV	Extracellular vesicle
mRNA	Messenger RNA
miRNA	microRNA
ncRNA	Non-coding RNA
NGS	Next generation sequencing
QC	Quality control
qPCR	Quantitative PCR
sEV	Small extracellular vesicle
TBS	Tris-buffered saline
UMIs	Unique molecular indies
