# Supplementary material for: Optimizing Small RNA Sequencing for Salivary Biomarker Identification: A Comparative Study of Library Preparation Protocols
Source: Int J Mol Sci. 2025 Nov 26;26(23):11437. doi: 10.3390/ijms262311437 (PMC12692187; doi:10.3390/ijms262311437)
Supplement: Supplementary file 1 [file ijms-26-11437-s001.zip › ijms-3975083-supplementary.pdf]

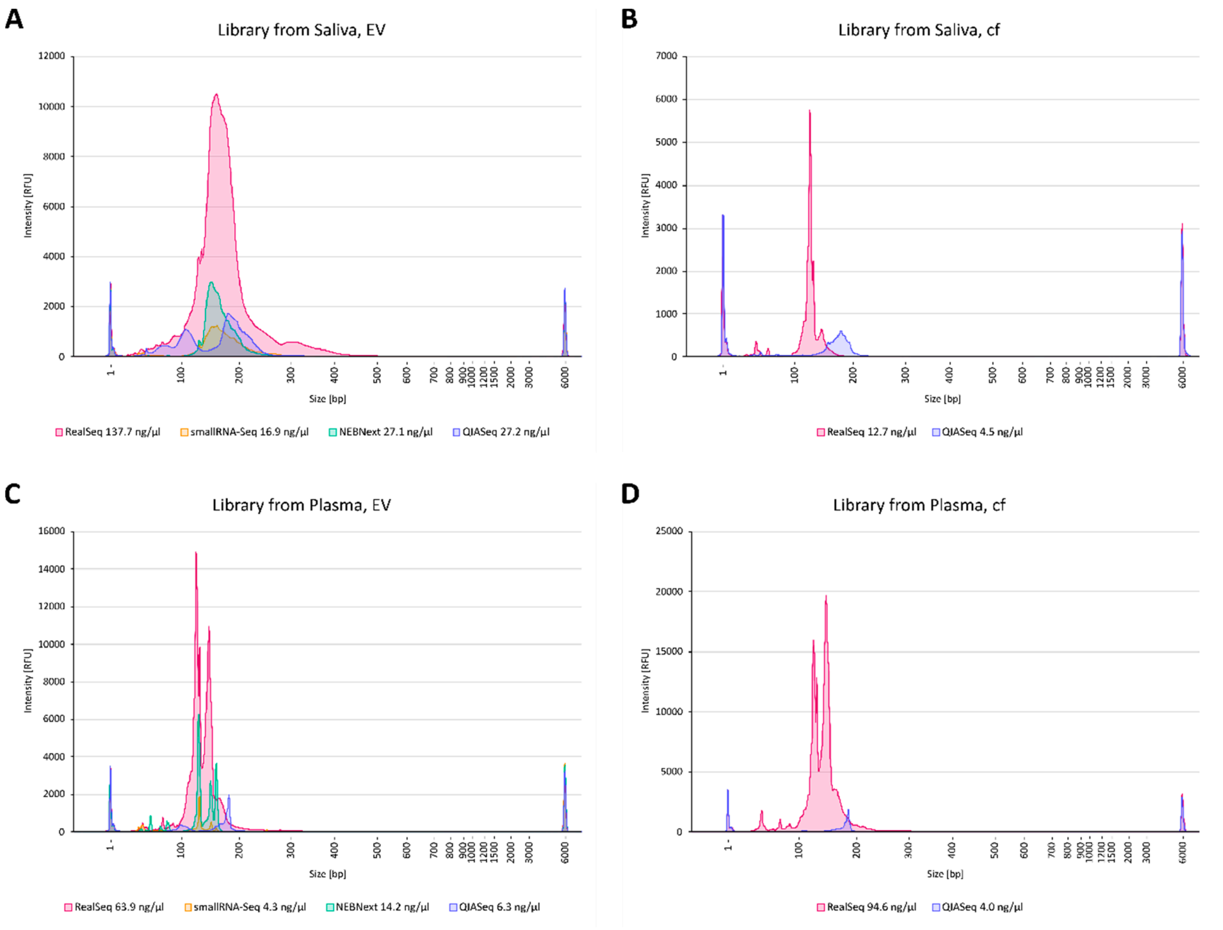

Supplementary Figure S1: Quality Control of the library amplicons using the fragment analyzer. The library preparation for small RNA was performed according to the manufacturer's manuals. For each preparation, a 1:10 dilution of amplified sample was analysed with the HS NGS fragment kit (1-6000 bp) applied on the 5300 Fragment Analyzer system (Agilent). The electropherogram shows peaks generated from saliva, EV (A); saliva, cf (B); plasma, EV (C); and plasma, cf (D) obtained by every library preparation kit. For RealSeq (magenta), smallRNA-Seq (orange) and NEBNext (green), the peaks about 150-160 bp correspond to the miRNA library and about 120-130 bp represents the adapter-dimers. For QIASeq (purple), the peaks about 180 bp correspond to the miRNA library and about 160 bp represents adapter-dimers.

A

	SALIVA, EV			SALIVA, CF	
	QIASEQ	REALSEQ	NEBNEXT	QIASEQ	REALSEQ
	MAPPED READS, total (%)				
miRNA	13,424 (15.32)	10,440 (24.26)	20,733 (31.84)	44,744 (52.42)	12,748 (43.18)
miscRNA	221 (0.25)	473 (1.10)	1,458 (2.24)	470 (0.55)	162 (0.55)
Mt-r/tRNA	236 (0.27)	653 (1.52)	238 (0.37)	247 (0.29)	189 (0.64)
rRNA	0 (0.00)	902 (2.10)	0 (0.00)	0 (0.00)	6,862 (23.24)
Other sRNA	267 (0.30)	780 (1.81)	22 (0.03)	564 (0.66)	2,564 (8.68)
discarded	73,504 (83.86)	29,778 (69.21)	42,668 (65.52)	39,328 (46.08)	6,998 (23.70)

B

	PLASMA, EV			PLASMA, CF	
	QIASEQ	REALSEQ	NEBNEXT	QIASEQ	REALSEQ
	MAPPED READS, total (%)				
miRNA	1,359,691 (98.33)	455,008 (95.22)	744,993 (84.64)	2,819,653 (96.54)	1,193,882 (89.82)
miscRNA	6,429 (0.46)	5,189 (1.09)	119,461 (13.57)	25,395 (0.87)	42,708 (3.21)
Mt-r/tRNA	2,331 (0.17)	3,276 (0.69)	511 (0.06)	43,326 (1.48)	56,349 (4.24)
rRNA	20 (0.001)	2,030 (0.42)	252 (0.03)	11 (0.00)	9,466 (0.71)
Other sRNA	1,111 (0.08)	1,338 (0.28)	1,047 (0.12)	2,449 (0.08)	1,806 (0.14)
discarded	13,234 (0.96)	10,992 (2.30)	13,974 (1.59)	29,957 (1.03)	25,020 (1.88)

Supplementary Figure S2: Composition of small RNA library in saliva and plasma.

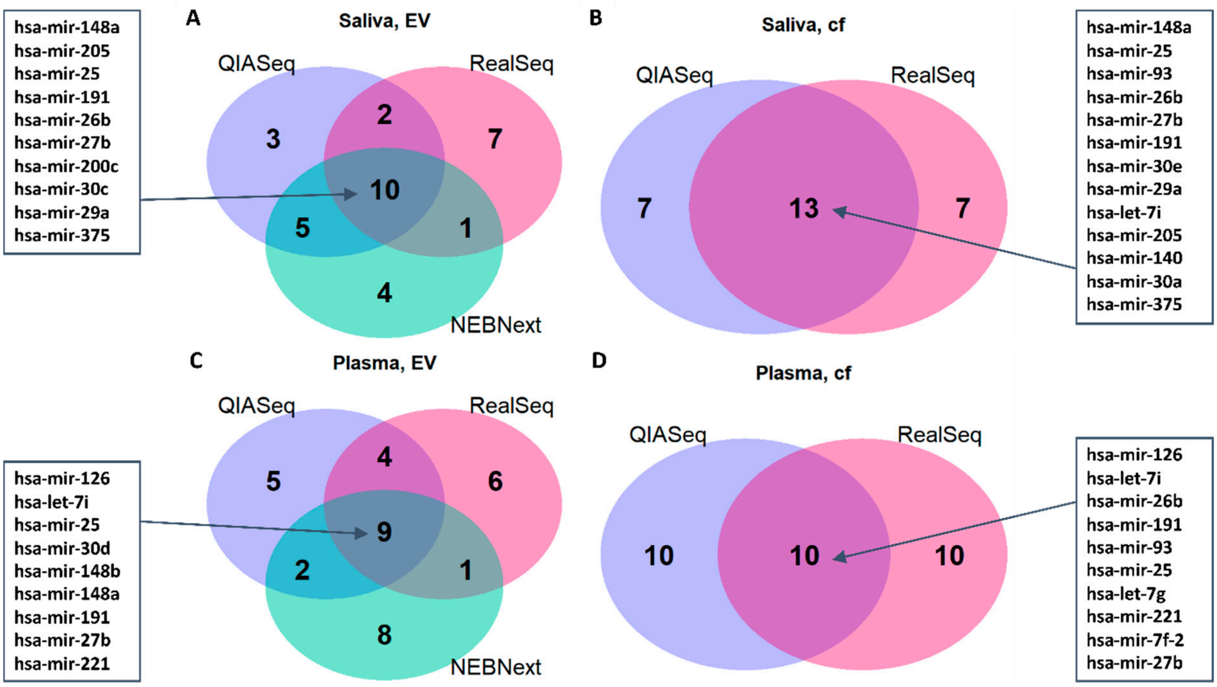

Supplementary Figure S3: Overlaps of Top20 miRNA of extracellular vesicles and cell-free plasma and saliva. The overlap of all detected miRNAs between each technique are illustrated using Venn diagrams plotted for (A) saliva, EV; (B) saliva, cf; (C) plasma, EV and (D) plasma, cf. The lists indicate the most frequent miRNA detected in parallel with all library preparation kits.

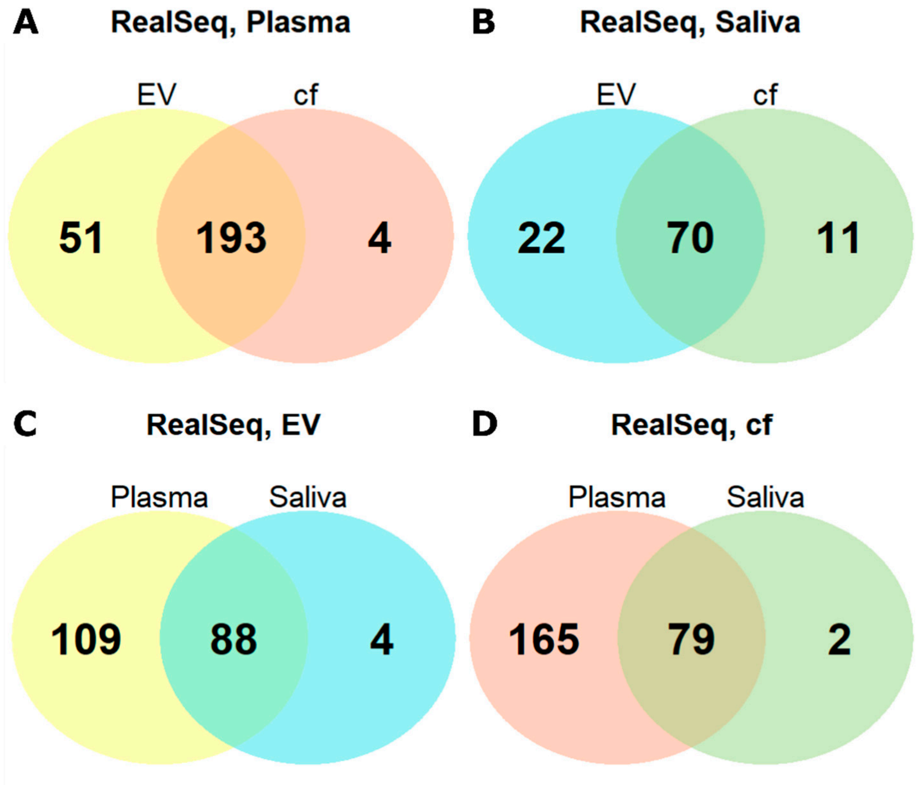

Supplementary Figure S4: Overlaps of miRNA profile in respect of sample matrices. The overlap of miRNAs between the different sample matrices detected by RealSeq are illustrated using Venn diagrams plotted for (A) RealSeq, Plasma; (B) RealSeq, Saliva; (C) RealSeq, EV and (D) RealSeq, cf.

A

	SALIVA, EV						SALIVA, CF			
	QIASEQ		REALSEQ		NEBNEXT		QIASEQ		REALSEQ	
	fraction (%)	SD	fraction (%)	SD	fraction (%)	SD	fraction (%)	SD	fraction (%)	SD
Total reads	100	0.00	100	0.00	100	0.00	100	0.00	100	0.00
Adapter trimming	75.59	7.64	79.72	1.07	64.93	0.87	63.42	0.00	18.89	4.10
Mapped reads	0.60	0.12	0.24	0.04	0.36	0.04	1.37	1.79	0.29	0.11
miRNA mapping	0.09	0.02	0.05	0.00	0.10	0.00	0.66	0.59	0.11	0.03

B

	PLASMA, EV						PLASMA, CF			
	QIASEQ		REALSEQ		NEBNEXT		QIASEQ		REALSEQ	
	Fraction (%)	SD	Fraction (%)	SD	Fraction (%)	SD	Fraction (%)	SD	Fraction (%)	SD
Total reads	100	0.00	100	0.00	100	0.00	100	0.00	100	0.00
Adapter trimming	73.08	3.21	48.70	3.68	50.96	2.84	88.08	4.98	61.79	3.94
Mapped reads	10.99	0.70	4.04	0.51	5.43	0.24	19.48	1.81	11.97	1.24
miRNA mapping	10.64	0.45	3.52	0.29	4.26	0.11	18.58	1.18	10.09	0.49

C

	MIRXPLORE UNIVERSAL REFERENCE					
	QIASEQ		REALSEQ		NEBNEXT	
	Fraction (%)	SD	Fraction (%)	SD	Fraction (%)	SD
Total reads	100	0.00	100	0.00	100	0.00
Adapter trimming	99.16	2.39	95.49	0.35	98.63	0.49
Mapped reads	35.75	1.26	31.48	2.34	36.84	3.48
miRNA mapping	34.61	0.81	31.02	0.13	36.32	2.42

Supplementary Figure S5: Summary check. The tables contain the corresponding data for the bar plots in Figure 7. They are intended to illustrate the sequencing efficiency by showing the mean percentage of reads after adapter trimming and genome miRNA mapping per library preparation kit.
